# Recommendations for ibrutinib treatment in patients with atrial fibrillation and/or elevated cardiovascular risk

**DOI:** 10.1007/s00508-019-1534-1

**Published:** 2019-08-14

**Authors:** Markus C. Stühlinger, Ansgar Weltermann, Philipp Staber, Daniel Heintel, Thomas Nösslinger, Michael Steurer

**Affiliations:** 1 University Clinic of Internal Medicine III/Cardiology and Angiology, Medical University of Innsbruck, Anichstraße 35, 6020 Innsbruck, Austria; 2 Zentrum für Tumorerkrankungen, Ordensklinikum Linz, Linz, Austria; 3 University Clinic of Internal Medicine I, Hematology and Hemostaseology, Medical University of Vienna, Vienna, Austria; 4 1st Medical Department, Center for Oncology and Hematology, Wilhelminenspital Vienna, Vienna, Austria; 5 3rd Medical Department, Hematology and Oncology, Hanusch Hospital Vienna, Vienna, Austria; 6 University Clinic of Internal Medicine V, Hematology and Oncology, Medical University of Innsbruck, Innsbruck, Austria

**Keywords:** BCR inhibitor, CLL, Chronic lymphocytic leukemia, Anticoagulation, Bleeding, BCR Inhibitor, CLL, Chronische lymphatische Leukämie, Antikoagulation, Blutung

## Abstract

Ibrutinib is the first clinically approved inhibitor of Bruton’s tyrosine kinase, an enzyme that is essential for survival and proliferation of B‑cells by activating the B‑cell receptor signalling pathway. Ibrutinib has been shown to be highly effective in B‑cell malignancies in clinical trials and is recommended in current international guidelines as a first and/or second line treatment of chronic lymphocytic leukemia. The drug has a favorable tolerability and safety profile but the occurrence of specific side effects (e.g. atrial fibrillation, bleeding and hypertension) may complicate or be of concern for doctors and patients considering the use of this treatment. In many cases, however, it is not necessary to withhold this effective therapy. In contrast, ibrutinib treatment can be initiated or continued, if certain recommendations are followed. The possibilities of prevention, diagnosis and management of specific clinical situations are discussed in detail and recommendations are derived, which should facilitate ibrutinib use.

## Zusammenfassung

Ibrutinib ist der erste zum klinischen Einsatz zugelassene Inhibitor der Bruton-Tyrosinkinase, eines Enzyms, das durch Aktivierung des B‑Zell-Rezeptor-Signalwegs für das Überleben und die Proliferation der B‑Zellen von wesentlicher Bedeutung ist. Ibrutinib hat in klinischen Studien hohe Wirksamkeit bei B‑Zell-Malignomen gezeigt und wird in aktuellen internationalen Leitlinien als Erst- und/oder Nachfolgetherapie zur Behandlung der chronischen lymphatischen Leukämie empfohlen. Grundsätzlich mit günstigem Verträglichkeits- und Sicherheitsprofil ausgestattet, kann jedoch das mögliche Auftreten spezifischer Nebenwirkungen (Vorhofflimmern, Blutungen und Hypertonie) den Einsatz dieser effektiven Therapie erschweren oder verhindern. In vielen Fällen ist es aber nicht notwendig, auf die Ibrutinib-Therapie zu verzichten – unter Berücksichtigung bestimmter Vorgaben kann diese fortgesetzt werden. Die Möglichkeiten von Prävention, Diagnose und Umgang mit konkreten Situationen werden in der Folge ausführlich behandelt, und es werden daraus Empfehlungen abgeleitet, welche die Entscheidung für das jeweilige Vorgehen erleichtern sollen.

## Ibrutinib—mode of action and appropriate use

Treatment with novel B‑cell receptor (BCR) signalling inhibitors results in high response rates and long progression-free survival (PFS) in patients with various B‑cell malignancies, such as chronic lymphocytic leukemia (CLL), follicular lymphoma (FL), mantle cell lymphoma (MCL) and Waldenström’s macroglobulinemia (WM) [1]. Ibrutinib is a first-in-class inhibitor of Bruton’s tyrosine kinase (BTK), a molecule essential to BCR signalling via formation of an irreversible covalent bond with Cys-481 in the adenosine triphosphate(ATP)-binding domain [2, 3]. This mechanism prevents activation of pathways required for B‑cell survival and proliferation, such as the nuclear factor-kappa B pathway [4, 5]. Ibrutinib binds reversibly to related kinases, such as the tyrosine kinase expressed in hepatocellular carcinoma [6]. It also interferes with lymphocyte homing and chemotaxis, resulting in the phenomenon of redistribution lymphocytosis [7]. Inhibition of BTK in malignant B‑cells further diminishes proliferation, survival, adhesion and migration of the malignant B‑cells to the growth-promoting microenvironment [1, 4]. Ibrutinib is administered continuously as an oral medication with a toxicity profile that compares very favorably with conventional chemotherapy and chemoimmunotherapy. The drug has been shown to exhibit effectiveness in a variety of B‑cell malignancies, such as CLL, MCL, FL and WM [8]. In randomized phase III clinical trials ibrutinib monotherapy was more effective than chlorambucil in the first-line treatment of older patients (RESONATE-2) [9] and more effective than ofatumumab in previously treated adults (RESONATE) [10]. Furthermore, a combination of ibrutinib, bendamustine and rituximab was more effective in previously treated adults than bendamustine plus rituximab in a phase III placebo-controlled study (HELIOS) [11]. In all these trials ibrutinib regimens displayed significantly better PFS, overall response rates and overall survival (OS) than the comparators. This benefit was seen regardless of adverse prognostic factors, such as del(17p)/TP53 and del(11q) mutations [5]. Updated safety and efficacy results of the RESONATE trial with up to 4 years of follow-up indicated that ibrutinib conveys sustained PFS and OS benefits regardless of high-risk cytogenetics [12]. Long-term follow-up of the RESONATE-2 study demonstrated continued greater and sustained improvements in patient reported outcomes (PRO) with ibrutinib as compared to chlorambucil [13]. A cross-trial comparison between single-agent ibrutinib treatment (derived from RESONATE-2) and chemoimmunotherapy regimens from published phase 3 studies showed that single-agent ibrutinib was associated with longer PFS and a generally more favorable safety profile despite longer treatment duration and a much longer collection period for adverse events. It is suggested that ibrutinib may potentially eliminate the need for chemotherapy in some patients with treatment naïve CLL [14]. However, despite its high efficacy and favorable toxicity profile, there is room for improvement to optimize ibrutinib treatment in clinical practice. In order to overcome potential obstacles and to achieve best possible patient outcomes, several attempts have been made to identify the most important practical issues and to propose relevant management recommendations to maximize the clinical benefits by using the drug in the safest, most appropriate way [15].

## Dosing, adjustments and severity grades of adverse events

The recommended starting dose of ibrutinib for the treatment of CLL and WM is 420 mg (3 capsules taken at the same time once daily), and for MCL the recommended dose is 560 mg (4 capsules) given continuously until disease progression or until unacceptable toxicity. As efficacy has been established at 420 mg in CLL, and the occurrence of adverse events (AE) can generally not be predicted in the individual patient, there is no evidence to support initiating ibrutinib at a lower dose, unless there is the possibility of a drug-drug interaction as discussed later.

**Fig. 1:**
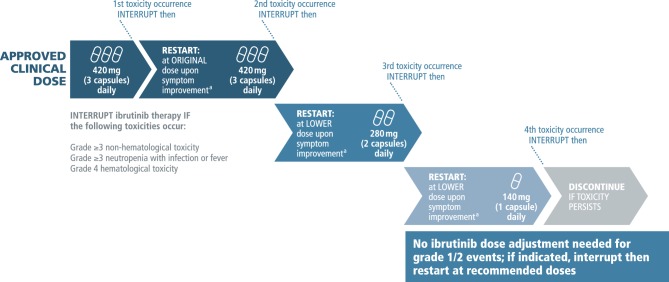
Ibrutinib dosing scheme. ^a^Resolution to grade 1 or baseline. Note: dose shown is for chronic lymphocytic leukemia (*CLL*); mantle cell lymphoma (*MCL*) dosing is 560 mg (4 capsules) daily. Source: Imbruvica®. Summary of product characteristics, 2017 [17]

Subgroup analyses from clinical trials suggest that variable ibrutinib dosing may impact PFS [16]. Therefore, it is recommended that the dose of ibrutinib should generally not be interrupted or reduced unless there is a valid clinical reason to do so. If interruption or dose modification are deemed necessary, adjustments should only be made in line with the algorithm provided in the ibrutinib summary of product characteristics (Fig. 1) that was applied in randomized trials with favorable clinical outcomes [17].

For the grading of toxicities or side effects leading to ibrutinib dose adjustments a well-known classification system is recommended and published in the Common Terminology Criteria for Adverse Events (CTCAE) [18]. The CTCAE displays grades 1 through 5 for each AE based on a general guideline as follows: Grade 1—mild (asymptomatic or mild symptoms; intervention not indicated); grade 2—moderate (minimal, local or noninvasive intervention indicated); grade 3—severe or medically significant but not immediately life-threatening (hospitalization or prolongation of hospitalization indicated; disabling); grade 4—life-threatening consequences (urgent intervention indicated); grade 5—death related to an adverse event.

It is noteworthy that no dose adjustment of ibrutinib is needed for adverse events in grades 1 and 2. For events in grade 3 or higher, treatment should be interrupted until toxicity resolves to grade 1 or baseline. Importantly, ibrutinib should be reinitiated at the same dose for the first occurrence of an AE (or at a reduced rate in the case of recurrence of events), if and as soon as it is deemed possible by the treating physician. It is crucial to go through the full cycle of dose interruptions before withdrawing ibrutinib to provide the patient with the best optimal treatment outcome (Fig. 1).

## Special considerations in individual patients

Ibrutinib is a continuous fixed-dose treatment, which is continued until disease progression or intolerance. Adverse events mostly occur at CTCAE grades 1 and 2, soon after starting the treatment, and the incidence generally decreases over time [19]. Nevertheless, data from clinical trials suggest that ibrutinib is associated with an increased risk of atrial fibrillation (AF) as well as an increased rate of bleeding [10, 20]. The occurrence of these AEs can lead to situations which require more difficult clinical decision making [1]. Overall, however, most of these events can be managed effectively allowing continuous ibrutinib use for optimal patient outcome.

### Atrial fibrillation

Atrial fibrillation (AF) has been reported in patients treated with ibrutinib. Particularly patients with cardiac risk factors (e.g., hypertension, previous history of AF) and acute infections are more commonly affected by the arrhythmia. The underlying arrhythmogenic mechanisms are not fully understood. The most plausible explanation refers to ibrutinib-mediated inhibition of local cardioprotective pathways, in particular of the PI3K-Akt, a regulator of cardiac protection under stress conditions [5, 20]. The PI3K inhibition could result in AF by activation of late sodium channels (INa-L), at least after chronic drug exposure [21]; however, ibrutinib dose dependence of this effect has not been investigated so far [22, 23]. Likewise, an alleged increase of ventricular arrhythmia in patients taking ibrutinib still needs to be investigated in terms of causality [24]. Alternatively, structural remodelling and Calcium handling disorders in the atrium as well as reduced activity in tyrosine kinase pathways other than PI3K-Akt are considered as potential mechanisms [25, 26].

The incidence of AF is generally higher in older patients, likewise CLL incidence increases with age [27]; however, in clinical trials the incidence of tachyarrhythmia was higher in ibrutinib-treated groups compared to matched controls of a similar age with other CLL treatment, and this effect was associated with initiation of ibrutinib treatment [9–11, 28]. In a pooled analysis of 1505 patients across 4 ibrutinib clinical trials, most AF events occurred within the first 6 months after initiation of ibrutinib treatment and continued at a lower rate over time. At the time of initial follow-up (16.6 months) the incidence of AF in the ibrutinib arm was 6.5% versus 1.2% in the comparator arm. At a later follow-up (36 months) cumulative incidence increased to 10.4% in the ibrutinib arm. The median duration of AF was 3 days and the severity mostly grades 1 and 2. These events were generally manageable with additional drugs without further sequelae. Only 14.3% of patients who developed AF had to discontinue ibrutinib treatment, and the discontinuation rate subsequently decreased to 9% with extended follow-up (<1% of the total population). Moreover, approximately 50% of patients did not even need dose modifications or temporary interruptions [15].

Known risk factors for AF include valvular heart disease, hypertension, congestive heart failure, obstructive sleep apnea, obesity, diabetes mellitus, alcohol consumption and chronic kidney disease [20]. In a database from the Mayo Clinic, comprising follow-up on 2444 patients with newly diagnosed CLL, 6.1% of patients had a history of symptomatic AF. The remaining CLL patients were diagnosed with previously unknown tachyarrhythmia at a rate of 1% per year with a total of 11.6% experiencing AF over a median follow-up period of 59 months. Older patients, men and patients who suffered from valvular heart disease and hypertension were identified as being more at risk for developing new onset AF in this cohort [20].

This information provides the context for interpreting rates of AF in CLL patients treated with novel therapies. Treatment and prevention of complications of AF pose a clinical challenge in balancing thrombosis and bleeding risks in patients taking ibrutinib [29]. A major cause of morbidity and mortality in patients with AF is cardioembolic stroke and systemic embolism. The risk of embolic events can be estimated by CHADS_2_ and CHA_2_DS_2_-VASc scores [30]. The CHA_2_DS_2_-VASc, which stands for congestive heart failure/left ventricular dysfunction, hypertension, age ≥75 years (2 points), diabetes, stroke (2 points), vascular disease, age 65–74 years, and sex category (female) score is the best validated tool for evaluating stroke risk in patients with AF or atrial flutter. There is strong evidence that patients with a CHA_2_DS_2_-VASc risk score of ≥2 benefit from anticoagulation to prevent a stroke [31]. Moreover, increasing evidence also suggests that patients with AF and one other CHA_2_DS_2_-VASc risk factor (other than female gender) would also benefit from anticoagulation ([32]; Table 1). Individuals with a CHA_2_DS_2_-VASc score of 0 do not need antithrombotic therapy, whereas those with a CHA_2_DS_2_-VASc score of 1 should receive anticoagulation based on physician and patient preference [30].

**Table 1:** Congestive heart failure/left ventricular dysfunction, hypertension, age ≥75 years (2 points), diabetes, stroke (2 points), vascular disease, age 65–74 years, and female sex category (CHA_2_DS_2-_VASc) score for calculation of embolic events (Source: [42, 56])

Clinical risk factors for stroke, TIA and systemic embolism (CHA_2_DS_2_-VASc score)	
CHA_2_DS_2_-VASc risk factor	Points
*Congestive heart failure* Moderate or severe systolic dysfunction and/or recently decompensated heart failure with hospitalization	+1
*Hypertension* Resting blood pressure >140/90 mm Hg on ≥2 occasions or current antihypertensive treatment	+1
*Age 75 years or older*	+2
*Diabetes mellitus* Fasting glucose >125 mg/dL (>7 mmol/L) or treatment with an oral hypoglycemic agent and/or insulin	+1
*Previous stroke, TIA or thromboembolism*	+2
*Vascular disease* Previous myocardial infarction, peripheral artery disease or aortic plaque	+1
*Age 65–74 years*	+1
*Sex category (female)*	+1

Patients with symptomatic AF should also be treated with rhythm or heart rate control therapy. Beta blocker treatment should be initiated as soon as possible in the case of fast ventricular rates. Decisions about the choice of alternative rate treatment or antiarrhythmic therapy or cardioversion depends on concomitant cardiac disease and should be performed in agreement with a consulting cardiologist [15].

### Bleeding

Bruton’s tyrosine kinase has an important role in glycoprotein VI signalling, and inhibition of this enzyme has been shown to block collagen-mediated platelet aggregation. This on-target effect has been observed in vivo [33], specifically ibrutinib has been associated with an increased risk of any grade bleeding even in early phase clinical trials [3]. Most bleeding AEs observed in the clinical studies were graded CTCAE 1 or 2. They occurred early, did not require dose interruptions or dose modifications, improved and frequently decreased over time. The incidence of severe bleeding events was equally low, decreased over time and was observed significantly less frequently after the first 6 months of therapy [34]. Essentially, minor bleeding (grades 1–2), such as petechia, contusion, epistaxis and bruising, accounted for the majority of bleeding events in patients receiving ibrutinib, and ibrutinib-related hemorrhagic events were observed in patients both with and without thrombocytopenia [15]; however, major hemorrhage (CTCAE grade 3 or higher) also occurred in ibrutinib-treated patients in clinical trials, with an incidence of 1–7% in CLL patients [3, 9, 10, 35]. Postprocedural bleeding accounted for a large number of bleeding events in initial clinical trials [10, 11], leading to amendments in study protocols recommending withholding the drug for at least 3–7 days presurgery and postsurgery depending upon the type of surgery and the risk of bleeding.

As described ibrutinib is associated with an increased risk of AF, which often mandates anticoagulation therapy, leading to difficult clinical decision making and balancing stroke vs. bleeding risk [29, 36]. Nevertheless, anticoagulants or antiplatelets were used in 50% of patients in ibrutinib phase 3 clinical trials and (after adjusting for anticoagulant or antiplatelet exposure) the risk of major hemorrhage was still shown to be lower in ibrutinib groups compared to controls. Furthermore, bleeding leading to ibrutinib discontinuation was infrequent, occurring only in 1% of all ibrutinib-treated patients. In contrast, the rate of low-grade bleeding was elevated in patients treated with ibrutinib compared to comparator regimens, but essentially a minor bleeding event was not predictive of subsequent major hemorrhage [37]. Ibrutinib is currently not recommended in patients who require therapy with vitamin K antagonists (VKA) like warfarin, but patients treated with non-oral anticoagulants (heparin, low-molecular weight heparin, LMWH) or direct oral anticoagulants (DOAC, also known as non-vitamin K oral anticoagulants, NOAC) were not excluded in clinical trials. Likewise, antiplatelet agents, including aspirin, clopidogrel and non-steroidal anti-inflammatory drugs (NSAIDs) could be administered in ibrutinib trials; however, concurrent use of antiplatelet agents resulted in increased rates of bleeding [38]. Furthermore, increased bleeding risk has also been reported occasionally with other drugs known to inhibit platelet function, including vitamin E [9] and fish oil [17].

Several additional factors may explain the broad range of reported bleeding events in clinical trials. Variability in disease-associated platelet defects and severity of thrombocytopenia may also play a role. Patients with CLL, for instance, often have varying degrees of mild platelet dysfunction, which is difficult to measure in laboratory tests, but is exacerbated by ibrutinib. Some authors have suggested that interactions between potential baseline platelet abnormalities and ibrutinib-related effects may determine the risk of bleeding [39]. Indeed, ibrutinib has a direct antiplatelet effect, based on the inhibition of BTK signalling downstream from both the platelet collagen receptor glycoprotein (GP) VI and the von Willebrand factor receptor, GPIb-V-IX. These effects of ibrutinib on platelet function might lead to improved outcomes in patients with elevated cardiovascular risk, and it is possible that antiplatelet agents in patients receiving ibrutinib therapy could be discontinued. This strategy could subsequently hypothetically decrease cardiovascular events and bleeding; however, there is currently insufficient data to suggest that ibrutinib could indeed be used for primary or secondary cardiovascular risk reduction [29, 38].

Several scores have been developed to estimate the risk of bleeding in patients treated with oral anticoagulants [40]; however, the most commonly used HAS-BLED (hypertension, abnormal renal/liver function, stroke, bleeding history or predisposition, advanced age, drugs/alcohol) score (Table 2) has not been shown to reliably predict bleeding events in large clinical studies and a score ≥3 should not lead to anticoagulant therapy being withheld or withdrawn. Recently, it has been shown that quantitative assessment of ristocetin-induced platelet aggregation (RIPA) could be a practical tool to monitor and manage bleeding tendency and might thereby increase the safety of ibrutinib therapy [41].

**Table 2:** Hypertension, Abnormal renal/liver function, Stroke, Bleeding history or predisposition, Elderly / advanced age, Drugs/alcohol (HAS-BLED) score (Source: [56])

Clinical characteristics composing the HAS-BLED bleeding risk score		
	HAS-BLED risk factor	Points
H	Hypertension (resting blood pressure >160 mm Hg systolic)	+1
A	*Abnormal renal and liver function (1 point each)* Chronic dialysis, renal transplantation or serum creatinine >200 µmol/l chronic hepatic disease (e.g., cirrhosis or biochemical evidence of significant hepatic derangement)	+1 or +2
S	Stroke (history of stroke or TIA)	+1
B	Bleeding (bleeding history or predisposition of bleeding)	+2
L	Labile INRs (<60% time in therapeutic range)	+1
E	Age >65 years	+1
D	Drugs (antiplatelet agents, NSAR) or alcohol abuse (1 point each)	+1

The most important clinical risk factors for major bleeding are age, female gender, a history of major bleeding, anemia and impaired renal function. As most of these risk factors are non-modifiable, control of hypertension, and prevention of comedication predisposing to bleeding and alcohol abstinence should be emphasized [42].

### Drug-drug interactions (DDI)

Ibrutinib is metabolized in the liver, primarily by cytochromes P450 and 3A4 (CYP3A4). Since many other drugs are metabolized by these enzymes, there is a potential for DDI [43]. In a recent analysis of 118 patients with CLL who were being treated with ibrutinib, 64% of patients were found to be taking medications that could increase, and 3% of patients were found to be taking medications that could potentially decrease ibrutinib levels [44]. Since efficacy and safety of ibrutinib treatment depends on the drug’s plasma concentration, concomitant use of strong or moderate CYP3A4 inhibitors/inducers should be avoided if possible. Table 3 shows a list of common drugs that are strong or moderate CYP34A inducers/inhibitors.

**Table 3:** Cyp3A4 inhibitors (Source: Gribben et al. [15])

Drug class	Substrate
Strong CYP3A4 inhibitors*	
Antibacterials	Clarithromycin, telithromycin
Antidepressants	Nefazodone
Antimycotics	Itraconazole, ketoconazole
Antivirals	Indinavir, nelfinavir, ritonavir, saquinavir
Other	Cobicistat, buprenorphine/naloxone
***Moderate CYP3A4 inhibitors***	
Antibacterials	Ciprofloxacin, erythromycin
Antihypertensives/antiarrhythmics	Amiodarone, diltiazem, dronedarone, verapamil
Anti-emetics/antinauseants	Aprepitant
Antimycotics	Fluconazole, voriconazole
Antineoplastic agents	Crizotinib, imatinib
Antivirals	Amprenavir, atazanavir, darunavir, fosamprenavir
Other	Grapefruit juice
Strong/moderate CYP3A4 inducers*	
Antibacterials	Rifampicin
Anti-epileptics	Carbamazepine, phenytoin
Other	St John’s wort** (*Hypericum perforatum*)

*Not an exhaustive list of CYP3A4 inhibitors/inducers with potential for drug-drug interactions with ibrutinib. For a more comprehensive list, please visit http://medicine.iupui.edu/clinpharm/ddis/

**contraindicated

Readers are encouraged to consult the Summary of product characteristics of the European Medicines Agency or analyze most recent guidelines on management of ibrutinib DDI. Per Summary of product characteristics at the time of this publication, the risk/benefit of continuing concurrent therapy should be discussed with the patient and alternative options that do not interact with CYP3A4 should be explored, if co-administration of ibrutinib with strong or moderate CYP3A4 inhibitors is being considered [15].

If the benefit of ibrutinib therapy outweighs the risk and a decision is made to administer a concomitant moderate CYP3A4 inhibitor, the dose of ibrutinib should be reduced to 280 mg once daily (2 capsules), and patients should be closely monitored. In exceptional cases, where coadministration of a strong inhibitor of CYP3A4 cannot be avoided, the ibrutinib dose should be reduced to 140 mg once daily (1 capsule) or withheld for up to 7 days [17]. Close monitoring for toxicity (for duration of concomitant use) and modification guidance (see Table 1) are essential in the described situations; however, extended interruption or termination of treatment has been shown to be detrimental to patient outcome and is therefore not recommended [17].

Grapefruit and Seville oranges should be avoided during ibrutinib treatment, as these contain moderate inhibitors of CYP3A4. Moreover, concomitant use of strong or moderate CYP3A4 inducers (e.g., carbamazepine, rifampicin, and phenytoin) should also be avoided. Preparations containing St. John’s wort (*Johanniskraut*) are contraindicated during treatment with ibrutinib, as efficacy may be reduced. If a strong or moderate CYP3A4 inducer needs to be used, the patient should be closely monitored for lack of efficacy. Mild inducers may be used concomitantly with ibrutinib; however, patients should also be monitored [17].

There is in vitro evidence indicating that ibrutinib inhibits P‑glycoprotein (P-gp) and breast cancer resistance protein (BCRP); however, there are no data to suggest that this interaction has any clinical effect. To minimize the potential for interactions, it is recommended that P‑gp and BCRP substrates with a narrow therapeutic range, such as digoxin, methotrexate or rosuvastatin, should be administered at least 6 h before or after ibrutinib intake. Particularly for older patients, polypharmacy presents the potential for multiple interactions with antiarrhythmic drugs, anticoagulants, as well as NSAIDS, fish oil, etc. [5, 17]. This could also apply to dabigatran, as the prodrug, dabigatran etexilate, is a substrate for P‑gp. The effect of ibrutinib on intestinal P‑gp could potentially impact the conversion of prodrug to active drug during absorption and affect dabigatran levels [45].

## Practical guide for patients considered for or treated with ibrutinib

As discussed above some of the most important clinically relevant adverse events during therapy with ibrutinib are AF and minor or major hemorrhages. Bleeding is also a frequent and an important complication of elective or urgent surgery. For these reasons, prevention, diagnosis and management of these clinical situations are discussed in the following section.

### Cardiovascular risk stratification before initiation of ibrutinib treatment

The CLL primarily affects older patients, who regularly present with coexisting medical conditions and comedication in addition to disease-related immunosuppression and myelosuppression [46]. Consequently, knowing a patient’s history is extremely important. Specifically, prior response to therapy (including side effects), a full list of concomitant medications and comorbidities should be documented. Patients with hypertension, cardiovascular (CV) disease and increased risk of bleeding need special medical attention. Thus, CV assessments (blood pressure, heart rate, electrocardiogram) are the mainstay of diagnostics before initiating ibrutinib therapy. Particularly, modifiable risk factors for AF should be identified and treatment of the latter should be optimized: hypertension, heart failure, diabetes mellitus, overweight and obesity, excess alcohol consumption, valvular heart disease, chronic obstructive pulmonary disease and hyperthyroidism should be controlled and adequately treated. Furthermore, the CHA_2_DS_2-_VASc score should be calculated before ibrutinib therapy as well as on a yearly basis thereafter. Due to increased bleeding risk in patients with CLL and the platelet-inhibiting effect of ibrutinib, the potential need for anticoagulation and modifiable risk factors for bleeding, including blood pressure control and diagnosis of potential causes of anemia, should be addressed. A history of AF itself, however, should not mean refraining from ibrutinib treatment, as clinical trials have shown that the recurrence of arrhythmias during treatment with ibrutinib was indeed rare in patients with a history of AF [5].

Hypertension has not only been shown to be a risk factor for AF, but also for bleeding and for stroke [5, 20]. Thus, modification of ibrutinib dosing should be considered in the case of a new diagnosis of hypertension or worsening of established hypertension during therapy, as these conditions can be triggered by ibrutinib; however, as with other side effects, it is important to maintain the recommended ibrutinib dose to achieve optimal outcomes. Therefore, other treatment options to improve blood pressure control should be considered before interruption or dose reduction of ibrutinib. To minimize the risk of complications, the interaction of some commonly used antihypertensive drugs with ibrutinib by inhibition of CYP 3A4 (e.g. calcium channel blockers) should be considered [47]. Furthermore, causes of secondary hypertension need to be excluded. The occasional occurrence of arthralgia and myalgia as well as the treatment of these symptoms also require careful consideration. Whenever possible, low dose analgesics (e.g., paracetamol) should be selected as first-line treatment with the possibility of dose escalation if required in an individual patient. The NSAIDs carry an increased risk of hypertension and bleeding events. If a decision is made to use these drugs, agents that inhibit platelet function to a low degree (e.g., celecoxib) should be preferred. Low-dose opioids or anti-epileptics could be considered as alternative options [29].

#### Recommendations for basic assessment

**Fig. 2:**
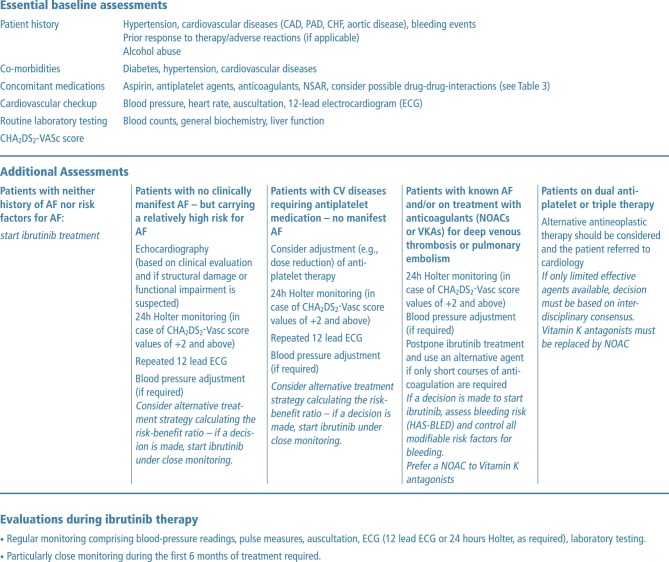
Basic and additional patient assessments before and during ibrutinib therapy

After initial assessment, regular clinical evaluation is recommended including pulse, heart rate, blood pressure measurements and auscultation, particularly within the first 6 months. Routine laboratory testing, including full blood counts, hemoglobin and general biochemical tests, should also be performed on a regular basis. Stroke risk should be estimated and documented by means of the CHA_2_DS_2_-VASc score before treatment and then every 12 months, even in those patients without clinically identifiable AF, to be prepared for informed anticoagulant management in case of incidence of this event. (Fig. 2, baseline assessments).

#### Patients with no history of AF and no associated risk factors

Basic assessments should be performed as discussed above. The CHA_2_DS_2-_VASc score should be calculated and documented. In the absence of any detectable risk factors ibrutinib therapy can be started.

#### Patients without AF but with elevated risk for AF

In addition to the basic assessments shown in Fig. 2, patients with increased AF risk and known structural heart disease or cardiovascular symptoms should undergo echocardiography and an examination by a cardiologist.

The CHA_2_DS_2_-VASc score should be calculated and, if the score is ≥2, a 24 h Holter monitoring should be performed to look for asymptomatic AF. If no AF is detected, repeated 12-lead electrocardiography (ECG) should be performed during follow-up. As with any patient, blood pressure control should be achieved and maintained during ibrutinib therapy. If asymptomatic or symptomatic AF is detected, oral anticoagulation is recommended in cases of elevated CHA_2_DS_2_-VASc score.

#### Patients with CV disease (without AF) requiring antiplatelet medication

All diagnostic and therapeutic measures should be carried out as discussed in the previous sections. If a decision is made to start ibrutinib therapy, comedication with antiplatelet agents (aspirin and/or clopidogrel) should be re-evaluated. It is tempting to be confident of the antiplatelet activity of ibrutinib and discontinue aspirin in an effort to reduce the risk for bleeding; however, at this point there are not sufficient clinical data to support this approach. In patients with elevated CV risk, who had prior myocardial infarction, bypass surgery, stroke or another CV event, combining ibrutinib with a single antiplatelet agent is a valid strategy. In most current recommendations aspirin at a maximum dose of 75–100 mg is preferred, as higher doses or clopidogrel would entail an increased bleeding risk while only achieving questionable additional clinical benefit [29, 48, 49]. Similarly to all other patient groups, blood pressure control is of major importance.

#### Patients treated with anticoagulants (DOAC or VKA) for stroke prevention in atrial fibrillation or for venous thrombosis or pulmonary embolism

Postponing ibrutinib therapy should be considered in patients who require anticoagulation for a limited duration (3–6 months), if this is deemed feasible. An alternative antineoplastic agent could be considered until the start of ibrutinib therapy, based on assessment of each individual risk-benefit for bleeding versus antineoplastic treatment efficacy [29]. Concurrent antiplatelet therapy in combination with anticoagulants and ibrutinib should be avoided unless there is a strong indication.

For patients requiring extended anticoagulation (>3–6 months), use of an agent other than ibrutinib or using a DOAC could be considered depending on the benefit-risk assessment for the individual patient. As described above additional antiplatelet agents should be avoided unless strongly indicated.

If a decision is made to start treatment with ibrutinib, the bleeding risk must be assessed (clinically or by HAS-BLED score), and all modifiable risk factors for bleeding should be addressed, including adequate blood pressure control and treatment of anemia. If a DOAC is administered, prescribing the lower available dose should be considered. The DOAC levels can be monitored with respect to possible pharmacological interactions. In this context, it is also important to remember that some cardiovascular drugs (verapamil, diltiazem, amiodarone) could cause DDIs with DOACs and/or ibrutinib.

As for the use of VKAs in a real-world setting, maintaining stroke risk patients within the therapeutic range of stable international normalized ratio (INR) can be difficult to achieve [50]. Even more important, VKA agents (e.g., warfarin) are not recommended for coadministration with ibrutinib by the European Medicines Agency (EMA) [17]. Some patients with high risk of stroke may be unable to receive oral anticoagulation. These patients should be alternatively treated with LMWH, which can be combined with ibrutinib. In general, ibrutinib should only be prescribed for these patients after a risk-benefit calculation and consideration of all alternative treatment options.

#### Patients who require dual antiplatelet or triple therapy (dual antiplatelet therapy and oral anticoagulation)

Combined anticoagulation and antiplatelet treatment during ibrutinib use should be avoided. If dual antiplatelet therapy (DAPT) or triple therapy is required, alternative antineoplastic therapy should be considered, if available, because of the high risk of major bleeding under ibrutinib [1]. If there are no alternative antineoplastic agents available, the patient should be referred to the cardiology department and managed according to an interdisciplinary consensus [29]. If it is decided to start or continue ibrutinib, VKA needs to be replaced by a DOAC, and single antiplatelet therapy in combination with a DOAC instead of triple therapy should be considered.

Delaying or interrupting ibrutinib therapy during a short course of DAPT should be considered; however, replacement of ibrutinib with an alternative antineoplastic agent is indicated if extended DAPT is deemed necessary [15, 29].

### Management of side effects during ibrutinib therapy

#### Atrial fibrillation

Patients who develop symptoms of arrhythmia (e.g. palpitations) or new onset dyspnea, dizziness, chest discomfort or fainting should be clinically evaluated. Moreover, a 12-lead ECG needs to be performed immediately. If AF is diagnosed, triggers of the arrhythmia, such as myocardial ischemia, hypertension, thyroid disorders, infection, sleep apnea, and electrolyte imbalance should be excluded or adequately managed. Beta blocker therapy should be initiated and the advice of a cardiologist should be sought and echocardiography performed. For patients who develop AF, ibrutinib should either be maintained or interrupted – based on the severity of the event – and restarted as soon as the heart rate is stabilized. Based on the evidence from clinical trials, it is recommended that patients are kept on ibrutinib whenever possible ([17]; see Fig. 1); however, alternative treatment strategies should be planned or considered if symptoms of AF cannot be controlled and the risk-benefit ratio dictates that ibrutinib needs to be discontinued. Patients receiving concomitant oral anticoagulation should be followed closely, particularly during the early phase of treatment. Stroke risk should be evaluated and compared with bleeding risk on an individual basis to determine whether continuation of anticoagulation is appropriate. If anticoagulation therapy is deemed necessary based on the risk of stroke (CHA_2_DS_2_-VASc score) and bleeding (HAS-BLED score or clinical risk factors), a DOAC is preferred over a VKA because of the lower risk of major bleeding events and because of the favorable stroke risk-benefit profile of DOACs in AF patients [30, 40, 51]. The DOACs have a relatively short half-life and rapid action. Compared to warfarin, their anticoagulant effect is more predictable and stable and is less influenced by diet and comedication, so that laboratory monitoring, and dose adjustments are not necessary in most cases. As each individual DOAC displays different additional advantages, it is not possible to give absolute consensus recommendations for a specific DOAC. Apixaban, for example, is characterized by an excellent gastrointestinal side effect profile. Dabigatran, on the other hand, offers the availability of an antidote and shows reduced potential for CYP3A4 interactions [15]. All factor Xa inhibitors show a favorable balance between efficacy and safety compared to VKA [51]. The DAPT or triple therapy with concomitant ibrutinib should be avoided. If this concomitant therapy is necessary, alternative anti-neoplastic treatment should be considered. Appropriate rate control of the arrhythmia should be started as soon as possible. The potential pharmacological interactions with P‑glycoprotein substrates (e.g., digoxin, dabigatran), CYP3A4-inhibiting anti-arrhythmic drugs (e.g., verapamil, amiodarone) and certain DOACs (e.g., apixaban, rivaroxaban) should be considered [44, 52]. AF was frequently low grade CTCAE and of short duration in clinical trials, therefore the risks and benefits of a rhythm control strategy should only be considered after repeated AF episodes and in highly symptomatic patients. If rhythm control is used, amiodarone, diltiazem and verapamil are best avoided due to drug interactions. If one of these agents is used, the dose of ibrutinib should be reduced as described in the DDI section. Electrical cardioversion may be prudent for symptomatic persistent AF failing rate control with beta blockers.

#### Bleeding

**Fig. 3:**
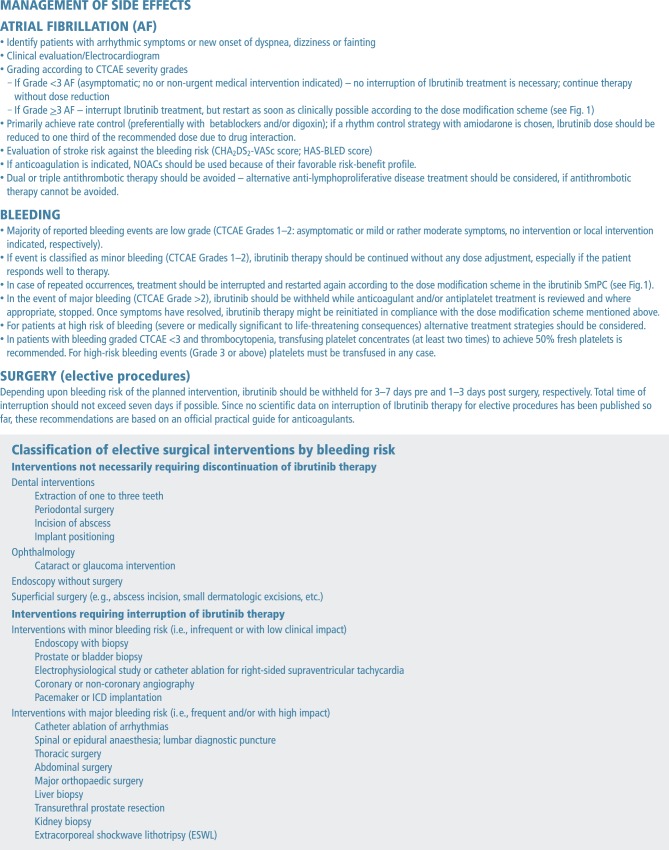
Ibrutinib-related adverse event management. Source: Heidbuchel et al. [57]

Low-grade bleeding can be managed with supportive care or by withholding ibrutinib for a short period of time [29]. Minor bleeding, however, should not be a reason to stop or reduce the dose of ibrutinib, particularly, if the patient is responding well to ibrutinib treatment [15]. In the case of recurrent events, ibrutinib dose should be reduced as recommended in Fig. 1. In the event of a major bleeding, steps should be taken to understand the underlying cause. If an association with ibrutinib is suspected, it is recommended that ibrutinib is interrupted while further investigations are performed. Coadministration of anticoagulant and antiplatelet treatment should be reviewed and stopped if deemed appropriate. Once bleeding is stopped and potential causes resolved, ibrutinib therapy may be reinitiated at the starting dose. If the toxicity reoccurs, daily dose should be reduced by 140 mg. A second reduction of the dose by 140 mg may be considered as needed. Ibrutinib therapy should be discontinued only if bleeding events persist or recur following two dose reductions (Fig. 1; [17]). In patients admitted for non-central nervous system (CNS) major bleeding, or those requiring transfusions, stopping ibrutinib and transfusing platelets is recommended, even in patients who are not thrombocytopenic [15, 29]. In vitro data suggest that transfusion of platelets to achieve 50% fresh platelets should correct hemostasis [53]. There are no strong recommendations in the event of major CNS bleeding, and these should be assessed on a patient-by-patient basis; however, generally, platelet transfusions are not advised for these bleeding events ([29]; Fig. 3).

#### Management of ibrutinib for elective and urgent surgical procedures

For patients undergoing surgery, ibrutinib should be withheld for 3–7 days before and 1–3 days after surgery, depending on the intervention and the patient-dependent risk of bleeding. Concomitant medications should also be reviewed and interrupted based on the risk of bleeding. In the event of urgent or emergency surgery, platelet transfusion (to receive 50% fresh platelets) should be performed [29, 53]. After surgery, the decision when to restart ibrutinib should be discussed with the treating surgeon. If it is deemed impossible to restart ibrutinib, a suitable alternative should be explored (Fig. 3).

## Conclusion

Ibrutinib is a paradigm shifting agent in a variety of B‑cell malignancies. It has shown high efficacy in treatment naïve CLL patients as well as relapsed or refractory CLL patients leading to significant improvements in OS and PFS. Consequently, current EU and US guidelines recommend ibrutinib for the first- and/or subsequent-line treatment for patients with CLL [54, 55].

Despite high clinical efficacy and an overall favorable toxicity profile, the possibility of the occurrence of side effects (AF, bleeding, hypertension) as well as the necessarily careful selection of treatment options for these events could pose some obstacles to the administration of ibrutinib. By providing a local perspective and recommendations for management of the adverse events associated with ibrutinib, we hope to give additional guidance and enable clinicians and patients alike to achieve the optimal outcome with this effective antineoplastic therapy.
